# AdipoRon Engages Microglia to Antinociception through the AdipoR1/AMPK Pathway in SNI Mice

**DOI:** 10.1155/2023/7661791

**Published:** 2023-04-10

**Authors:** Qian Fang, Jie Li, Yaping Wang, Xinli Liu, Yu Shi, Jiali Chen, Hongrui Zhan, Yanyan Zeng, Wen Wu

**Affiliations:** ^1^Department of Rehabilitation, Zhujiang Hospital, Southern Medical University, Guangzhou, 510282 Guangdong, China; ^2^Guangdong-Hong Kong-Macao Greater Bay Area Center for Brain Science and Brain-Inspired Intelligence; Key Laboratory of Mental Health of the Ministry of Education; Guangdong Province Key Laboratory of Psychiatric Disorders, Southern Medical University, Guangzhou, 510515 Guangdong, China; ^3^Department of Rehabilitation, The Fifth Affiliated Hospital of Sun Yat-sen University, Zhuhai, 519000 Guangdong, China

## Abstract

**Background:**

Microglia-associated neuroinflammation plays a crucial role in the initiation and development of neuropathic pain (NeuP). AdipoRon is an analog of adiponectin that exerts an anti-inflammatory effect in various diseases through the adiponectin receptor 1 (AdipoR1) signaling mechanism. Adenosine monophosphate-activated protein kinase (AMPK) is a downstream target of AdipoR1, and the AdipoR1/AMPK pathway is involved in the regulation of inflammation. This study is aimed at investigating whether AdipoRon could alleviate NeuP by inhibiting the expression of microglia-derived tumor necrosis factor-alpha (TNF-*α*) through the AdipoR1/AMPK pathway.

**Methods:**

In vivo, the NeuP model was established in mice through the spared nerve injury. The von Frey test was used to detect the effect of AdipoRon on the mechanical paw withdrawal threshold. Western Blot was performed to detect the effects of AdipoRon on the expression of TNF-*α*, AdipoR1, AMPK, and p-AMPK. Immunofluorescence was performed to observe the effects of AdipoRon on spinal microglia. In vitro, lipopolysaccharide (LPS) was used to induce inflammatory responses in BV2 cells. The effect of AdipoRon on cell proliferation was detected by CCK-8. qPCR was used to examine the effects of AdipoRon on the expression of TNF-*α* and polarization markers. And the effect of AdipoRon on the AdipoR1/AMPK pathway was confirmed by Western Blot.

**Results:**

Intraperitoneal injection of AdipoRon alleviated mechanical nociception in SNI mice, and the application of AdipoRon reduced the expression of TNF-*α* and the number of microglia in the ipsilateral spinal cord. Additionally, AdipoRon decreased the protein level of AdipoR1 and increased the protein level of p-AMPK in the ipsilateral spinal cord. In vitro, AdipoRon inhibited BV2 cell proliferation and reversed LPS-induced TNF-*α* expression and polarization imbalance. Furthermore, AdipoRon reversed the LPS-induced increase in AdipoR1 expression and decrease in p-AMPK expression in BV2 cells.

**Conclusions:**

AdipoRon may alleviate NeuP by reducing microglia-derived TNF-*α* through the AdipoR1/AMPK pathway.

## 1. Background

Neuropathic pain (NeuP) can be induced by a lesion or disease of the somatosensory nervous system caused by trauma, metabolic disorders, viral infections, autoimmune disorders, or chemotherapy. The disease is characterized by spontaneous pain, hyperalgesia, and allodynia and has a prevalence of 6.9%-10% in the general population [1, 2]. Chronic neuropathic pain is a suffering for patients and a burden for the medical system and society, making it necessary to unravel the pathological mechanisms [3]. Growing evidence has shown that neuroinflammation is a crucial factor in the initiation and development of NeuP [4]. And microglia have an important role in the regulation of neuroinflammation [5]. Proinflammatory microglia (M1 phenotype) express markers such as CD32 and tend to synthesize proinflammatory substances to promote neuroinflammatory responses. In contrast, anti-inflammatory microglia (M2 phenotype), characterized by molecules such as CD206, produce anti-inflammatory substances to inhibit the progression of neuroinflammation [6]. An animal study showed that more microglia reach a proinflammatory state than an anti-inflammatory state in the spinal cord of rats during the later stage of chronic constriction injury, suggesting an imbalance in microglial M1-M2 polarization in neuropathic pain [7]. Therefore, targeting microglia to modulate neuroinflammation may be a feasible strategy for the treatment of NeuP.

Adiponectin, a hormone secreted by adipocytes, inhibits inflammation in a variety of diseases through the adiponectin receptor 1 (AdipoR1) signaling mechanism [8]. Recent studies have shown that knockout of adiponectin reduces the paw withdrawal threshold in wild-type mice, while intrathecal injection of adiponectin attenuates the inflammatory pain-like behavior induced by complete Freund's adjuvant in rats [9, 10]. These studies suggest that adiponectin may be involved in the regulation of pain. Unfortunately, the biological properties of adiponectin limit its clinical application [11]. AdipoRon is an analog of adiponectin with the ability to activate AdipoR1 [12]. Numerous studies have confirmed that AdipoRon plays an anti-inflammatory role in various diseases [13–15]. However, the effect of AdipoRon on NeuP has not been investigated.

Adenosine monophosphate-activated protein kinase (AMPK), a downstream target of AdipoR1, plays an essential role in physiological processes such as glucose metabolism, lipid metabolism, and protein synthesis. Activation of AdipoR1 promotes the conversion of AMPK to phosphorylated AMPK (p-AMPK) [16, 17]. Studies have shown that the AdipoR1/AMPK pathway is abnormal in diseases such as diabetic nephropathy and intracerebral hemorrhage, and treatments targeting this pathway have been confirmed to be effective [18, 19]. Recombinant CTRP9 improved neurological function, reduced brain edema, and alleviated inflammation through the AdipoR1/AMPK pathway after intracerebral hemorrhage [20]. AdipoRon improved cognitive dysfunction in a mouse model of Alzheimer's disease by activating the AdipoR1/AMPK pathway [21]. However, it is unclear whether AdipoRon can target the AdipoR1/AMPK pathway to produce beneficial effects on NeuP.

In this study, we aimed to investigate whether AdipoRon could alleviate NeuP and inhibit microglia-associated TNF-*α* expression and to explore the role of the AdipoR1/AMPK pathway in the antinociception of AdipoRon.

## 2. Materials and Methods

### 2.1. Animals

Male C57BL/6 J mice (6-8 weeks) were purchased from the Zhuhai BesTest Bio-Tech Co., Ltd. (Zhuhai, China). The animals were kept at 5-6 per cage under controlled temperature (24 ± 1°C) and light-dark cycle (darks on from 7: 00 pm to 7: 00 am) with free access to food and water. Before being included in the experiment, these animals were given at least four days to acclimatize to the environment. All experiments were approved by the Animal Ethics Committee of Zhujiang Hospital of Southern Medical University on January 26, 2021 (approval number: LAEC-2020-153FS). Each effort was made to minimize the number and suffering of experimental animals.

### 2.2. The Neuropathic Pain Model

The NeuP model was induced by the spared nerve injury (SNI) in mice as previously described [22, 23]. Briefly, the mice were anesthetized by intraperitoneal injection (i.p.) of tribromoethanol (400 mg/kg). Then, a 5–millimeter incision was made on one thigh to expose the sciatic nerve and its three branches. Next, the tibial and peroneal nerves were ligated and transected, and the sural nerve was preserved. At last, the muscle and skin were closed in sequence. For Sham surgery as a control, mice receive the same manipulation except for nerve ligation and nerve transection.

### 2.3. Drugs

AdipoRon was purchased from MedChemExpress (USA) and dissolved in a mixed solvent (dimethyl sulfoxide, polyethylene glycol 300, Tween-80, and saline were mixed at a ratio of 1 : 4 : 0.5 : 4.5). After surgery, the mice were randomly divided into four groups: Sham group, SNI group, SNI+Vehicle group, and SNI+AdipoRon group. Mice in the SNI+AdipoRon group were given AdipoRon by intraperitoneal injection at 50 mg/kg every 24 hours for a total of five injections. And mice in the SNI+Vehicle group were injected intraperitoneally with volume-matched solvent as a control.

### 2.4. von Frey Test

The von Frey test was used to determine the mechanical paw withdrawal threshold in mice. The mice were placed individually in test cages with metal mesh floors and allowed to acclimate to their surroundings for at least 30 minutes before testing. The von Frey filaments of 0.04, 0.07, 0.16, 0.4, 0.6, 1.0, 1.4, 2.0, and 4.0 g were applied to the skin on the lateral side of the sole of the paw for 5 seconds. The response was considered positive if the mice exhibited any nocifensive behavior. All tests started at 0.6 g. If a positive response occurred, the adjacent smaller filament was used the next time, and if a negative response occurred, the adjacent larger filament was used the next time. There was a 30-second interval between stimuli. Testing continued until four responses were collected after the first response change. For data processing, the Up-Down Reader software was used [24]. The final results were converted to a 50% paw withdrawal threshold (50% PWT) for comparison using Dixon's up-down method [25].

### 2.5. Western Blot

The protein was extracted from the L4-L6 segments of the ipsilateral spinal cord, separated with SDS-PAGE gel (10% and 12%), and then transferred onto polyvinylidene fluoride membranes. After blocking the nonspecific binding sites with 5% skimmed milk solution for 2 h at room temperature, the membranes were incubated with the primary antibodies anti-AdipoR1 (1 : 1000, Abcam, ab126611), anti-AMPK (1 : 1500, Proteintech, 66536-1-Ig), anti-p-AMPK (1 : 1000, Cell Signaling Technology, #2535), anti-TNF-*α* (1 : 1000, Sangon Biotech, D221347-0025), anti-GAPDH (1 : 10000, Proteintech, 60004-1-Ig), and anti-*β*-actin (1 : 2000, Abcam, ab8227) for 12-16 h at 4°C. Then, the membranes were incubated with the corresponding secondary antibodies (1 : 5000, Solarbio) for 1.5 h at room temperature. The signal density of the primary antibody was visualized with an imaging system and quantified with ImageJ.

### 2.6. Immunofluorescence

Mice were deeply anesthetized and the L4-L6 segments of the spinal cord were collected after transcardial perfusion with saline followed by 4% paraformaldehyde. The tissues were fixed in 4% paraformaldehyde at 4°C for 12 h and dehydrated in 30% sucrose at 4°C for 3-4 days. Then, the 15-micrometer-thick sections of spinal samples were prepared using a cryostat. After blocking with 10% bovine serum albumin and 0.3% Triton X-100 in phosphate-buffered saline for 2 h at room temperature, sections were incubated with primary antibodies against AdipoR1 (1 : 1000, Abcam, ab126611), Iba-1 (1 : 800, Wako, 011-27991), GFAP (1 : 300, Cell Signaling Technology, #3670), and NeuN (1 : 800, Millipore, MAB377) for 16-20 h at 4°C. After washing, sections were incubated with corresponding secondary antibodies (1 : 500, Bioss) at room temperature for 1.5 h. Fluorescent staining results were observed and photographed using a Nikon microscope. And the number of spinal microglia was counted using ImageJ.

### 2.7. Cell Culture

BV2 cells, a mouse microglia cell line, were cultured in high-glucose DMEM containing 10% fetal bovine serum and 1% penicillin/streptomycin in a 5% CO_2_ incubator. BV2 cells were stimulated with lipopolysaccharide (LPS) at a concentration of 100 ng/ml or AdipoRon at a concentration of 50 *μ*M for 24 h.

### 2.8. Cell Counting Kit-8 (CCK-8)

The effect of AdipoRon on the proliferation of BV2 cells was assayed by CCK-8. BV2 cells were plated in 96-well plates and treated with AdipoRon at a concentration of 50 *μ*M for 24 h. Afterwards, CCK-8 solution was added to the 96-well plate and incubated for 1-3 h. The proliferation of cell was determined by measuring the OD value at 450 nm.

### 2.9. Quantitative Real-Time PCR (qPCR)

Total RNA was isolated from BV2 cells using Trizol (Accurate Biology, China) and quantified by a NanoDrop spectrophotometer (Thermo Fisher Scientific, USA). Then, mRNA was reverse-transcribed into cDNA using the Evo M-MLV Reverse Transcription Kit (Accurate Biology, China). And each target gene was quantified by the ABI qPCR system (Thermo Fisher Scientific, USA) using the SYBR Green PCR Master Mix (Accurate Biology, China). The following Primers were used: TNF-*α* (sense primer: 5′-TCA CTG GAG CCT CGA ATG TC-3′; antisense primer: 5′-TCT GTG AGG AAG GCT GTG CA-3′), CD32 (sense primer: 5′-AGG GCC TCC ATC TGG ACT G-3′; antisense primer: 5′-GTG GTT CTG GTA ATC ATG CTC TG-3′), CD206 (sense primer: 5′-CTC TGT TCA GCT ATT GGA CGC-3′; antisense primer: 5′-CGG AAT TTC TGG GAT TCA GCT TC-3′), and GAPDH (sense primer: 5′-AGG TCG GTG TGA ACG GAT TTG-3′; antisense primer: 5′-TGT AGA CCA TGT AGT TGA GGT CA-3′). GAPDH was used as control. Results were normalized to GAPDH and calculated using the comparative CT method.

### 2.10. Statistical Analysis

All data in this study were expressed as mean ± SEM. Date were analyzed, compared, and visualized with the GraphPad Prism 7.0 software. Comparisons between multiple groups were performed using one-way ANOVA. Behavioral scores were analyzed by two-way repeated-measures ANOVA. Tukey's multiple comparison was used as a post hoc analysis. *P* values less than 0.05 were considered statistically significant.

## 3. Results

### 3.1. AdipoRon Alleviated Mechanical Nociception in SNI Mice

**Figure 1:**
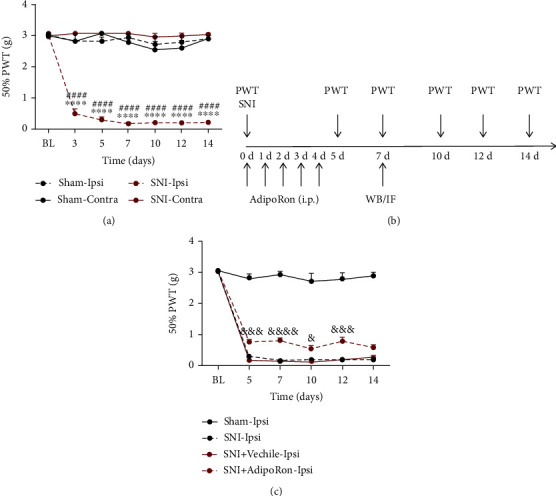
AdipoRon increased 50% PWT in SNI mice. (a) The 50% PWT in the ipsilateral (Ipsi) and contralateral (Contra) hind paw of Sham or SNI mice before and after surgery. (b) Timeline of in vivo experiments. (c) The ipsilateral 50% PWT in SNI mice at all time points after intraperitoneal injection of AdipoRon or Vehicle. All results were presented as mean ± SEM (*n* = 6 per group). ^∗∗∗∗^*p* < 0.0001 vs. SNI-Contra, ^####^*p* < 0.0001 vs. Sham-Ipsi, and ^&^*p* < 0.05, ^&&&^*p* < 0.001, and ^&&&&^*p* < 0.0001 vs. SNI+Vehicle-Ipsi.

To verify the validity of the NeuP model, the von Frey test was performed before and after surgery to obtain 50% PWT. In the SNI group, the ipsilateral 50% PWT decreased significantly from postoperative day 3 compared to the contralateral 50% PWT, but there was no such difference in the Sham group. Also, the ipsilateral 50% PWT in the SNI group was significantly reduced after surgery compared to the ipsilateral 50% PWT in the Sham group (Figure 1(a)). These results indicate that the NeuP model was successfully established in this study.

Then, the effects of AdipoRon on SNI mice were examined according to the timeline in Figure 1(b). The behavioral results showed a significant rebound in the ipsilateral 50% PWT in the SNI+AdipoRon group from day 5 to day 12 after SNI compared to the ipsilateral 50% PWT in the SNI+Vehicle group (Figure 1(c)). These data suggest that AdipoRon is able to alleviate mechanical nociception in SNI mice.

### 3.2. AdipoRon Downregulated TNF-*α* and Microglia Numbers in the Spinal Cord of SNI Mice

**Figure 2:**
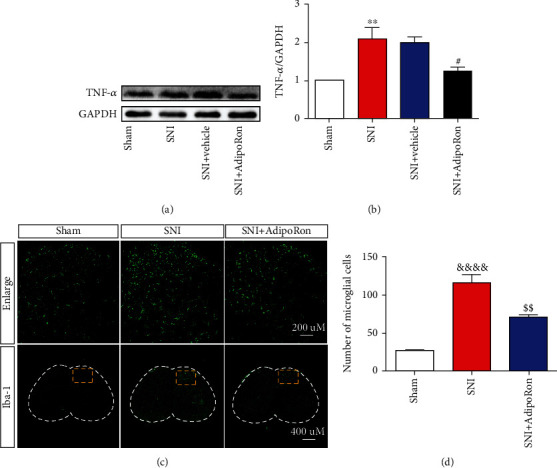
AdipoRon reduced the protein expression of TNF-*α* and the number of Iba-1-positive cells in the ipsilateral spinal cord of SNI mice. (a, b) Representative blots and quantification of the effect of AdipoRon on the TNF-*α* protein expression in the ipsilateral spinal cord of mice on day 7 after SNI. (c) Representative fluorescence images of microglia in the ipsilateral SDH (spinal dorsal horn) of mice on day 7 after SNI. Scale bar: 400 *μ*M and 200 *μ*M. (d) Summarized data for microglia numbers in the ipsilateral SDH from the three groups shown in (c). Data were presented as mean ± SEM (*n* = 4 per group). ^∗∗^*p* < 0.01 vs. Sham-Ipsi, ^#^*p* < 0.05 vs. SNI+Vehicle-Ipsi, ^&&&&^*p* < 0.0001 vs. Sham, and ^$$^*p* < 0.01 vs. SNI.

Microglia-associated neuroinflammation is an essential pathological cause of NeuP. Therefore, we obtained spinal cord tissue for testing on day 7 after surgery. The result of Western Blot showed that the protein level of TNF-*α* in the ipsilateral spinal cord was significantly increased in the SNI group compared to the Sham group. At the same time, immunofluorescence showed a marked increase in the number of microglia in the ipsilateral spinal dorsal horn (SDH) of the SNI group compared with the Sham group. After the application of AdipoRon, the protein level of TNF-*α* in the ipsilateral spinal cord was significantly lower in the SNI+AdipoRon group than in the SNI+Vehicle group, while the number of microglia in the ipsilateral SDH was also significantly reduced (Figures 2(a)–2(d)). Collectively, these results demonstrate that AdipoRon downregulates the number of microglia and the protein level of TNF-*α* in the ipsilateral spinal cord of SNI mice.

### 3.3. AdipoRon Alleviated Mechanical Nociception via the AdipoR1/AMPK Pathway

**Figure 3:**
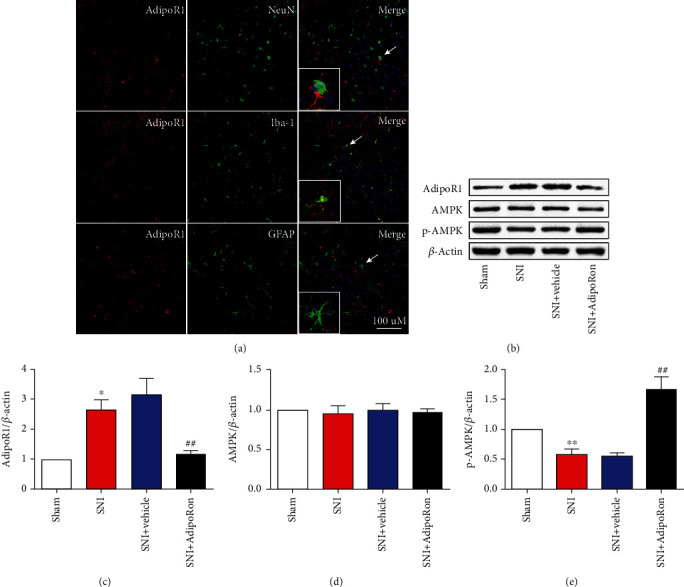
AdipoRon regulated the AdipoR1/AMPK pathway in the ipsilateral spinal cord of SNI mice. (a) Representative fluorescence images of ipsilateral SDH showing double staining for AdipoR1 (red) and Iba-1 (green), GFAP (green), and NeuN (green), respectively. Scale bar: 100 *μ*M. (b–e) Representative Western Blots and quantification of AdipoR1, AMPK, and p-AMPK in the ipsilateral spinal cord of Sham, SNI, SNI+Vehicle, and SNI+AdipoRon on day 7 after surgery. Data were presented as mean ± SEM (*n* = 4 per group). ^∗^*p* < 0.05 and ^∗∗^p < 0.01 vs. Sham-Ipsi. ^##^*p* < 0.01 vs. SNI+Vehicle-Ipsi.

To explore the mechanism underlying the antinociceptive and anti-inflammatory effects of AdipoRon, firstly, we examined the cellular localization of AdipoR1 in the SDH. We observed that AdipoR1 was mainly localized in microglia and neurons (Figure 3(a)).

Next, Western Blot was used to detect the expression of the AdipoR1/AMPK pathway in the ipsilateral spinal cord on day 7 after surgery. As shown in Figures 3(b)–3(d), compared with the Sham group, the protein expression of AdipoR1 was increased, but the expression of p-AMPK, a downstream molecule of AdipoR1, was inhibited in the ipsilateral spinal cord of the SNI group. After treatment with AdipoRon, the protein expression of AdipoR1 was significantly reduced, and the protein expression of p-AMPK was significantly increased in the ipsilateral spinal cord of the SNI+AdipoRon group, compared to the SNI+Vehicle group. Together, these data suggest that the antinociceptive and anti-inflammatory effects of AdipoRon may be related to the AdipoR1/AMPK pathway.

### 3.4. AdipoRon Inhibited TNF-*α* Expression by Regulating the Proliferation and Polarization of BV2 Cells

**Figure 4:**
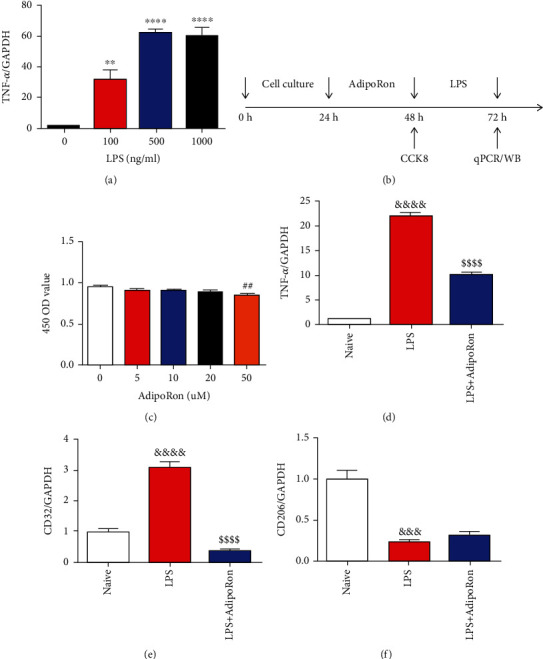
AdipoRon inhibited LPS-induced TNF-*α* expression in BV2 cells. (a) Effects of different concentrations of LPS on the expression of TNF-*α* in BV2 cells. (b) Timeline of in vitro experiments. (c) Effects of different concentrations of AdipoRon on the proliferation of BV2 cells. (d) AdipoRon at a concentration of 50 *μ*M inhibited the expression of TNF-*α* induced by LPS in BV2 cells. (e, f) Effects of AdipoRon at a concentration of 50 *μ*M on the LPS-induced polarization imbalance in BV2 cells. All results were presented as mean ± SEM (*n* = 3). ^∗∗^*p* < 0.01, ^∗∗∗^*p* < 0.001, and ^∗∗∗∗^*p* < 0.0001 vs. 0 ng/ml, ^##^*p* < 0.01 vs. 0 *μ*M, ^&&&^*p* < 0.001 and ^&&&&^*p* < 0.0001 vs. Naive, and ^$$$^*p* < 0.001 and ^$$$$^*p* < 0.0001 vs. LPS.

BV2 cells were used to further investigate the effect of AdipoRon on microglia-associated inflammation. LPS was applied to induce the inflammatory response in BV2 cells. To determine the stimulating concentration, BV2 cells were treated with 100 ng/ml, 500 ng/ml, and 1000 ng/ml of LPS for 24 h, respectively. The results of qPCR showed that all concentrations of LPS significantly increased the TNF-*α* mRNA level (Figure 4(a)). Therefore, 100 ng/ml was used as the stimulation concentration in subsequent experiments.

The effects of AdipoRon on BV2 cells were examined according to the timeline of Figure 4(b). First, we examined the effect of different concentrations of AdipoRon on BV2 cell proliferation. The results of CCK-8 showed that the 450 nm OD value of the 50 *μ*M group was significantly reduced compared to the 0 *μ*M group (Figure 4(c)). These results suggest that AdipoRon significantly inhibited the proliferation of BV2 cells at a concentration of 50 *μ*M. Next, we observed the effect of this concentration of AdipoRon on LPS-induced TNF-*α* mRNA expression in BV2 cells. As shown in Figure 4(d), compared with the LPS group, the mRNA expression of TNF-*α* was significantly decreased in the AdipoRon group. In addition, we also examined the effect of AdipoRon on the polarization of BV2 cells. The results of qPCR showed that the mRNA expression of CD32 was significantly increased, and the mRNA expression of CD206 was significantly decreased in the LPS group compared to the Naive group. The application of AdipoRon reversed the increase in CD32 mRNA expression induced by LPS (Figures 4(e) and 4(f)). The above results suggest that AdipoRon may inhibit LPS-induced TNF-*α* expression by affecting the proliferation and polarization of BV2 cells.

### 3.5. AdipoRon Inhibited TNF-*α* Expression in BV2 Cells via the AdipoR1/AMPK Pathway

**Figure 5:**
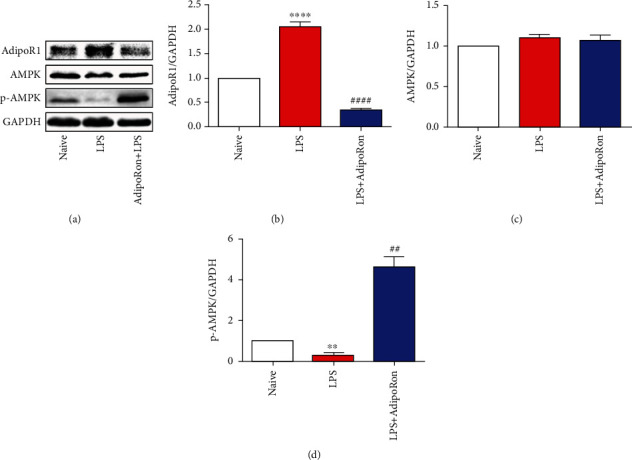
AdipoRon regulated the AdipoR1/AMPK pathway in LPS-treated BV2 cells. (a–d) Representative blots and quantification of AdipoR1, AMPK, and p-AMPK in BV2 cells in the Naive, LPS, and LPS+AdipoRon groups. Data were presented as mean ± SEM (*n* = 3). ^∗∗^*p* < 0.01 and ^∗∗∗∗^*p* < 0.0001 vs. Naive and ^##^*p* < 0.01 and ^####^*p* < 0.0001 vs. LPS.

We examined the AdipoR1/AMPK pathway on BV2 cells and found that compared with the Naive group, the protein expression of AdipoR1 was increased and the protein expression of p-AMPK was decreased in the LPS group. After pretreatment with AdipoRon, the protein expression of AdipoR1 was significantly reduced, while the protein expression of p-AMPK was significantly increased compared to the LPS group (Figures 5(a)–5(d)). These results were consistent with those observed in animals. Thus, these data imply that AdipoRon may inhibit TNF-*α* expression in BV2 cells via the AdipoR1/AMPK pathway.

## 4. Discussion

In this study, we explored the effects of AdipoRon on NeuP. We employed a spared nerve injury model to simulate mechanical pain in mice and used LPS to induce inflammatory responses in BV2 cells. Our results demonstrate that AdipoRon is able to alleviate mechanical nociception and inhibit spinal TNF-*α* expression in SNI mice and that these effects may be achieved by regulating the proliferation and polarization of microglia. In addition, our results also suggest that the AdipoR1/AMPK pathway may be involved in the regulation of AdipoRon on microglia. Therefore, this work is expected to provide new drug option and intervention target for the treatment of NeuP.

Microglia, the innate immune cells of the central nervous system, are involved in the regulation of neuroinflammation [26]. In recent years, growing evidence suggests that microglia play an important role in the pathology of NeuP [27]. Microgliosis and microglia activation occur after nerve injury, manifesting as the proliferation and release of bioactive mediators to facilitate pain signaling [28]. In our study, the number of microglia and the expression of TNF-*α* were increased in the spinal cord after SNI. Also, in vitro experiments showed high expression of TNF-*α* in BV2 cells after LPS stimulation. These results are consistent with the prevailing view that microglia-mediated neuroinflammation is involved in NeuP [29]. Importantly, the application of AdipoRon suppressed the increase in the number of microglia and the expression of TNF caused by SNI or LPS. Previously, a similar inhibitory effect was observed in Alzheimer's disease. The intensity of Iba-1 staining and the levels of IL-1*β* and TNF-*α* were obviously reduced in the brains of 5×FAD mice after AdipoRon treatment [30]. These results suggest that AdipoRon is able to inhibit inflammation by reducing the number of microglia. In addition, we observed a significant increase in CD32 mRNA (the marker of proinflammatory cells) and a significant decrease in CD206 mRNA (the marker of anti-inflammatory cells) in BV2 cells following stimulation with LPS. Microglia have a dual role in the regulation of neuroinflammation [31]. There is increasing evidence that modulating microglial polarization to restore the balance between proinflammation and anti-inflammation is more beneficial for the recovery of NeuP than blocking microglial activation or eliminating microglia. Activation of G protein-coupled receptors reduced pain-like behaviors by upregulating the IL-10/endorphin pathway in spinal microglia, but this effect was inhibited by minocycline, an inhibitor of microglial activation [32]. In our study, the mRNA level of CD32 was significantly decreased after AdipoRon pretreatment, while the mRNA level of CD206 was also slightly increased. And in a previous study, AdipoRon reduced the damage caused by intracranial hemorrhage via promoting the M2-type polarization of microglia [19]. These data suggest that AdipoRon dampens the inflammatory responses by regulating the polarization of microglia. Certainly, the concept of polarization has its limitations at this stage, and microglia may have a more diverse phenotype. However, using this concept in this work could help us to better understand the role of AdipoRon in the regulation of microglia-associated inflammation.

AdipoRon is a small-molecule AdipoR agonist. Recent studies have shown that AdipoRon exerts adiponectin-like effects in diseases such as diabetes and obesity and compensates for the limitations of adiponectin in clinical applications [33]. Knockout of adiponectin induced pain-like behaviors in normal mice, while regular swimming exercise relieved neuromaic nociception in rats accompanied by an increase in adiponectin level [9, 34]. These results suggest a link between adiponectin and pain modulation. In this study, the mechanical paw withdrawal threshold in mice was significantly decreased after SNI, but an intraperitoneal injection of AdipoRon moderated this phenomenon within a certain period of time. And AdipoRon inhibited the expression of TNF-*α* in both SNI mice and BV2 cells. TNF-*α* is a proinflammatory cytokine that is critical for the transition from acute pain to chronic pain. In the liver, AdipoRon attenuated carbon tetrachloride-induced liver damage by inhibiting the expression of proinflammatory cytokines and activation of hepatic stellate cells [14]. In the heart, AdipoRon relieved cardiopulmonary bypass-induced cardiac inflammation and functional impairment by inhibiting TLR4/TNF-*α* signaling in cardiomyocytes [13]. Therefore, these results suggest that AdipoRon alleviates mechanical nociception in SNI mice by inhibiting TNF-*α* expression in the spinal cord.

AdipoRs, mainly including AdipoR1 and AdipoR2, mediate the different functions of adiponectin [35]. Previous studies have shown that the anti-inflammation effect of adiponectin is mainly exerted through AdipoR1. The ability of adiponectin to inhibit the release of proinflammatory cytokines from A*β*O-treated BV2 cells was abolished after transfection with AdipoR1 siRNA but not AdipoR2 siRNA [36]. The expression level of AdipoR1 is different in different diseases [18, 19]. In our study, the expression of spinal AdipoR1 was increased on day 7 after SNI. This may be a compensatory increase associated with decreased levels of adiponectin in the nervous system following nerve injury. A study has shown that knockout of adiponectin downregulated the nociceptive threshold in normal mice, and the expression of spinal AdipoR1 increased on day 7 after the partial sciatic nerve ligation [9]. In addition, in a mouse model of Alzheimer's disease, the level of adiponectin in the cerebrospinal fluid decreased, while the expression of AdipoR1 in the brain increased [30]. AdipoR1 is an upstream molecule of AMPK that promotes the conversion of AMPK to p-AMPK [12]. In our study, the ratio of spinal p-AMPK/AMPK decreased significantly on day 7 after SNI. Inhibition of p-AMPK expression is associated with the occurrence of NeuP. In rats with chronic constrictive injury, an increase in nociceptive sensitivity was accompanied by a decrease in spinal p-AMPK expression [37]. These results suggest that the AdipoR1/AMPK pathway is altered in NeuP. AdipoRon has been shown to cross the blood-brain barrier [30]. Following treatment with AdipoRon, we observed a decrease in spinal AdipoR1 expression and an increase in spinal p-AMPK expression in SNI mice, accompanied by an improvement in mechanical nociception. Additionally, the results of immunofluorescence showed that AdipoR1 was expressed in spinal microglia. Then, we also observed a similar effect of AdipoRon on the AdipoR1/AMPK pathway in BV2 cells. These results suggest that the beneficial effects of AdipoRon on NeuP may be related to the AdipoR1/AMPK pathway.

There are still some limitations to the current study. (1) Immunofluorescence showed that AdipoR1 was localized to microglia and neurons in the spinal cord. It has been suggested that AdipoR1 is involved in the regulation of dopamine neuron activity and firing rate. However, the role of AdipoR1 in spinal cord neurons has not been explored in the present study. (2) We concentrated on the anti-inflammation of AdipoRon in SNI mice, but we did not investigate the other properties such as inhibition of insulin resistance or promotion of autophagy and whether AdipoR2 was involved. (3) We did not perform a knockdown to further validate the role of AdipoR1. (4) To avoid the influence of the estrous cycle of female animals on the results, male animals were selected as experimental objects. Whether there is a gender difference in the analgesic effect of AdipoRon was not investigated in this study. Further studies are needed to explore the analgesic mechanisms of AdipoRon.

## 5. Conclusions

**Figure 6:**
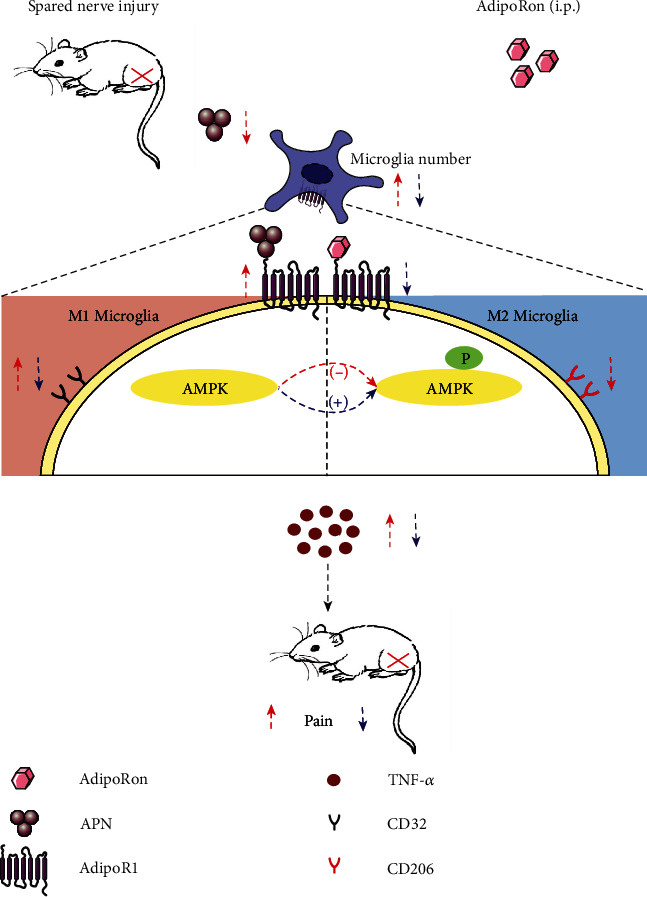
Schematic illustration of the analgesic effect of AdipoRon. AdipoRon reduced M1-type microglia through the AdipoR1/AMPK pathway to inhibit TNF-*α* expression and improve mechanical nociception. The red arrows represent the pathological process, and the blue arrows represent the AdipoRon effect.

In general, our findings suggest that AdipoRon attenuates mechanical nociception and inhibits spinal TNF-*α* expression in SNI mice and that these effects may be achieved through regulating the proliferation and polarization of microglia. Furthermore, our findings also suggest that the AdipoR1/AMPK pathway may be involved in the regulation of AdipoRon on microglia (Figure 6). Although the analgesic mechanism of AdipoRon remains to be further investigated, our findings provide a possible therapeutic option for relieving NeuP by inhibiting neuroinflammation.

## Data Availability

The data used to support the findings of this study are available from the corresponding authors upon request.
